# Supplementary material for: Transfer of learning: Analysis of dose-response functions from a large-scale, online, cognitive training dataset
Source: PLoS One. 2023 May 17;18(5):e0281095. doi: 10.1371/journal.pone.0281095 (PMC10191334; doi:10.1371/journal.pone.0281095)
Supplement: S1 Appendix — (PDF) [file pone.0281095.s001.pdf]

S1 Appendix. Descriptions of the CT games used in the present study

CT was provided via the Lumosity program, which included 69 different games across web and mobile apps over the time period in which the study data were collected. (Games were introduced and discontinued over time, so the exact number of available games varied.) Lumosity games are modeled on paradigms used to study specific cognitive functions in the lab or clinic. Based on their primary cognitive demands, they can be organized into seven cognitive domains: Memory, Attention, Flexibility, Problem Solving, Speed of Processing, Math, and Language. The majority of games, however, make demands in multiple cognitive domains (e.g., Processing Speed and Flexibility, or Attention and Memory).

Addition Storm (Math)

Addition Storm is similar to Raindrops (see below) but restricted to addition problems.

Assist Ants (Attention)

Assist Ants exercises divided attention and multiple object monitoring. In this game, users are required to track multiple moving ants and prevent them from colliding with each other or other obstacles by placing raindrop bumpers. Ants continually accrue seeds on their backs while they move around the screen and lose their seeds when they have collisions. There are 10 levels. New obstacles are introduced in the first 4 levels and then found in different combinations after that, giving users some variety in and across levels. The goal of the game is to maximize the number of seeds collected. Reaching a certain threshold number of seeds in a 60 second round will advance the user to the next level.

Birdwatching (Attention)

Birdwatching engages and exercises distributed and divided visual attention and speed of processing. This game challenges the user to identify a centrally presented letter while simultaneously indicating the position of a peripherally presented bird and a varying number of distractor objects. The visual system is challenged further by the introduction of complex backgrounds, picturing natural scenes. The user then engages in a secondary task in which a word must be constructed from the identified letters. The game advances to the next level when the user achieves a criterion level of performance. Each progressive level is marked by shorter presentations of central and peripheral targets and increasing numbers of distractor objects in the periphery.

Brain Shift (Flexibility)

Brain Shift exercises task switching. In this game, two cards are presented on the screen. On each trial, a number and a letter appear on one of the cards. If the stimulus appears on the top card, then the user should indicate “yes” if the number is even and “no” if the number is odd. On the other hand, if the stimulus appears on the bottom card, the user should indicate “yes” if the letter is a vowel and “no” if the letter is a consonant. After a number of correct responses, the challenge is increased by removing the prompt explaining the rules; thus, the user must remember the rules while accurately and rapidly indicating responses. The user is asked to respond quickly and accurately to as many stimuli as they can in a 60 second time period.

Brain Shift Overdrive (Flexibility)

Brain Shift Overdrive is very similar to Brain Shift (see above), but with increased challenge in task switching. In this version, there are four cards, arranged in a grid. If the number and letter appear in the upper left, then the correct response is to click “yes” when the number is even and “no” when the number is odd. If the number and letter appear in the lower left, then the correct response is to click “yes” when the number is odd and “no” when the number is even. The user is looking for a vowel in the upper right and a consonant in the lower right. The user is asked to respond quickly and accurately to as many stimuli as they can in a 60 second time period.

By the Rules (Problem Solving)

By the Rules exercises logical reasoning and working memory. In this game, users must identify the hidden rule in a dynamic card game by indicating whether each revealed card follows the rule. Rules involve features of the pattern depicted on the card, such as color, shape, and number. Exercising mental flexibility and working memory, the user is challenged to formulate hypotheses about what the current rule might be and then dynamically update that hypothesis as new information becomes available. The user is told when they have guessed a particular rule by correctly sorting a certain number of cards consecutively, and when the next rule is being presented. This type of reasoning ability involves the intersection of inductive and deductive reasoning, and in that way mimics the type of decision-making that happens in a wide variety of real-world contexts. Users try to determine as many rules as possible within a limited number of cards.

Chalkboard Challenge (Math)

Chalkboard Challenge exercises quantitative reasoning and calculation. In this game, users are presented with two arithmetic expressions, one on the left and one on the right, and must indicate which value is larger (or whether they are equal). The game rewards speed and accuracy. As users complete trials successfully, the arithmetic expressions become more complex, beginning with single numbers and progressing up to expressions including several integers, operators (addition, subtraction, multiplication, and division), and levels of parentheses. The user is asked to respond quickly and accurately to as many stimuli as they can in 50 seconds plus additional time awarded for correct answers.

Color Match (Flexibility)

Color Match exercises response inhibition and selective attention. This game is based on the Stroop Task, a classic test of semantic interference between color and meaning of words. Each trial shows two cards, each displaying a color word (black, red, blue, or yellow). The text displayed on the right card is presented in one of the four aforementioned colors while the text displayed on the left is presented in black. Users must read the word on the left card and indicate “yes” if the meaning of that word matches the color of the text on the right card and “no” if the color does not match. The user should ignore the meaning of the word displayed on the right card and only pay attention to its color. The goal is to make as many correct responses as possible per 45 second session, and users are additionally rewarded for completing sequential trials correctly.

Contextual (Language)

Contextual exercises reading comprehension and vocabulary knowledge. Each round consists of a short story or article with one passage of text presented at a time. Each passage contains one or more incorrectly used words. The user must click each incorrect word in the text and then choose a replacement from the sidebar. Game difficulty is adaptively adjusted along two scales, one measuring

user success identifying errors (comprehension), and the other their success selecting appropriate word replacements (vocabulary)

Continuum (Language)

Continuum exercises vocabulary knowledge. This game requires users to order 3 or 4 words that vary along a (semantic) continuum according to their order of magnitude (e.g., elder, adult, child). Each game consists of 12 trials. Difficulty (defined by list word frequency, length, and school-grade level) is adjusted trial-by-trial based on accuracy and speed. Game score is based on difficulty level, accuracy, and speed.

Disconnection (Flexibility)

Disconnection is similar to Disillusion (see below) except that the colors and shapes change in each session.

Disillusion (Flexibility)

Disillusion exercises task switching, response inhibition, and selective attention. In this game, users are presented with a grid of connected puzzle pieces, each marked with a shape and color. The user is presented with a single additional piece and must place it on the puzzle. The orientation of that piece indicates whether to match it to contiguous pieces based on the color attribute (if long dimension is along vertical axis) or shape attribute (if long dimension is along horizontal axis). A user is penalized for not matching a piece on the correct attribute. Users must learn the association and then place as many pieces as quickly and as accurately as possible in a 60 second time period.

Division Storm (Math)

Division Storm is similar to Raindrops (see below) but is restricted to division problems.

Eagle Eye (Attention)

Eagle Eye exercises integration of information across the visual field. This game is similar to Birdwatching (see above) but adapts to the user's performance within a session. The user must identify a centrally presented number or symbol while simultaneously indicating the position of a peripherally presented bird in the presence of distractors. The game increases in difficulty by shortening presentation time, increasing visual range, and increasing the number distractors.

Ebb and Flow (Flexibility)

Ebb and Flow exercises task switching, response inhibition, and selective attention. Each trial consists of a screen full of leaves all pointing in one direction and all traveling in one direction. The leaves are color coded and, depending on the color, the user's task is either to indicate the direction the leaves are pointing or the direction the leaves are moving. The response is a 4-alternative forced choice task (up, down, left, or right). Trials can either be congruous, in which the leaves are pointing in the same direction that they are moving, or incongruous, in which the leaves are pointing in a different direction from the direction of motion. The color of the leaves dictates the rule the user is asked to follow for that trial, with green meaning the user should indicate which direction the leaves are pointing and yellow meaning that the user should indicate which direction the leaves are moving. The leaf color (rule) is maintained for a random number of trials in order to build up an automatic response. When the leaf

color (i.e., rule) is switched, users must then switch their focus to the opposing attribute. Users are asked to respond to as many trials as quickly and accurately as possible in a 60 second time period.

Editor's Choice (Language)

Editor's Choice exercises vocabulary knowledge and fluency. This game involves the recognition of synonyms. On each trial, a list of words is shown with a target word at the top. The rest of the list contains either one or two synonyms of the target word, which the user is supposed to select as quickly as possible. Each game consists of 10 trials. Difficulty level is the school grade level of the words and is adjusted trial-by-trial based on the user's performance. Scoring is based on accuracy and difficulty level.

Face Memory Workout (Memory)

Face Memory Workout is an n-back visual working memory task and is similar to Memory Match (see below). Rather than matching abstract objects, however, users must recognize and match human faces. By responding accurately, users can progress from 1-back to 2-back to 3-back, meaning that users must match the current trial with the trial that appears one trial previously, two trials previously, or three trials previously. Game score is based on speed and accuracy.

Familiar Faces (Memory)

Familiar Faces exercises long-term and short-term memory. This game challenges the user's ability to create associations between visual and verbal information, such as associating a person's name with their face. In this game, the user is asked to remember names and food orders from multiple characters. The user is later asked to recall each character's name and to select their order from a list of options. In order to earn larger tips, the user must recall the names and orders accurately. As the user progresses by answering accurately, the game becomes more complicated, with more characters and more complex orders. Not only does the user need to remember names during a single session, they must also remember names from past sessions, mimicking the real life situation and challenging long term memory. Game score is tied to total tips collected.

Feel the Beat (Attention)

Feel the Beat exercises temporal attention and response inhibition. In this game, the user is presented with a drum pattern and then asked to imitate the pattern 5 times in order to establish a sense of its timing and rhythm. Auditory beats are accompanied by visual cues displayed as pink and blue icons (beats) that pulse sequentially in a clockwise direction around a pair of rings (representing a musical measure). The user must establish and maintain a rhythm that enables them to tap the right keys/buttons at the right times to match the pattern in each trial. The user's timing (how close they are to hitting the right buttons/keys at the right times) are translated into accuracy ratings, and users must achieve at least 80% accuracy in a play session in order to advance to a higher level.

Follow That Frog (Memory)

Follow That Frog exercises spatial working memory. This game challenges users to follow the hops of a frog across a pond filled with lily pads. The game is an adaptive spatial version of the n-back task in which users must indicate where the frog hopped a given number of hops back. This number increases with several successive successful trials and decreases after a mistake. Progress persists across sessions.

This game is a variation on n-back designed to be more interactive than the standard match/no match framework. The basic premise is a follow-the-leader task, where the user must follow the computer-controlled frog and jump on the same lily pads in the same order. In this variation, the n-back value is the number of moves the user is behind the computer-directed frog. Game score is based on accuracy and difficultly level (number of lily pads between the leader and follower).

Fuse Clues (Problem Solving)

Fuse Clues exercises logical reasoning, quantitative reasoning, and calculation. The main goal of this game is to develop the user's ability to recognize quantitative relationships or rules in number series. The game requires users to examine a sequence of numbers and generate a hypothesis about the relationship that exists between them. On each trial, some numbers in the sequence are left blank, and the user is asked to select an appropriate number to go in each blank space. Numbers are represented in the game as fuses that are plugged into an outlet to complete a circuit and charge a battery. A gameplay is composed of 4 trials and users advance adaptively from one trial to the next, moving up or down in level depending on their performance and speed of success. Game score is based on accuracy and level.

Halve Your Cake (Math)

Halve Your Cake exercises quantitative reasoning and calculation. This game challenges users to quickly compare and solve mathematical problems involving fractions that are presented in a variety of visual and symbolic forms. Users have a limited time period to solve as many math problems/comparisons as they can. Each problem represents a step in a recipe, and each completed recipe represents a different level. Problems increase in difficulty through a total of 12 levels, with each level culminating in the unveiling of a new baked creation. Game score is based on speed and accuracy.

Highway Hazards (Speed of Processing)

Highway Hazards exercises processing speed and divided attention. The goal of the game is to react quickly and efficiently to various road obstacles while going around a racetrack. The user’s speed increases as they dodge obstacles. Each obstacle is preceded by a warning sign indicating in which of the three lanes of the track it will appear. As the player goes faster, the warning signs appear for shorter amounts of time. Obstacles include rough patches of road and tumbleweeds. Additionally, the user must avoid cars coming in the opposite direction, which can be seen as they approach. Each game lasts approximately a minute and a half to two minutes. At that time, a finish line scene appears, along with a score based on the users speed and number of obstacles avoided.

Lost in Migration (Attention)

Lost in Migration exercises selective attention and response inhibition. This game utilizes a simple flanker task. Each trial consists of a presentation of five birds pictured in a pattern resembling a flock in flight. The user’s task is to indicate the direction of the middle bird. The response is a 4-alternative forced choice task. Trials can be either congruous, where the middle bird is pointed in the same direction as the other birds, or incongruous, where the middle bird is pointed in a different direction. The challenge of response inhibition occurs on the incongruous trials, when the user is tempted to indicate the direction of the majority of birds. The goal is to make as many correct responses as possible per 45 second session. Game score is based on speed and accuracy.

Magic Chance (Math)

Magic Chance exercises quantitative reasoning and calculation. The goal of this game is to analyze and evaluate probability questions presented using virtual cards. A game session includes 5 trials, and in each trial, users are given up to 2 tries to solve the probability question. The user responds to a question by placing cards with pictures of objects onto the table to achieve the desired distribution of object qualities described (e.g., chance of purple or star = 1/3). Object qualities differ in terms of shape and color. Correct answers trigger green check marks, wrong answers trigger red Xs. When two incorrect answers are given within a trial, the trial ends and users are shown an example of a correct answer. The game ends when 5 trials have been completed, either correctly or incorrectly. Difficulty level is adjusted adaptively, based on speed and accuracy. Game score is calculated from accuracy, speed, and difficulty level.

Masterpiece (Problem Solving)

Masterpiece exercises spatial reasoning. In this game, the user rotates and places puzzle pieces to fit together within a puzzle frame. Each game session is composed of 4 puzzles. These puzzles range from two to nine pieces. Score and difficulty level are based on the number of clockwise or counter-clockwise rotation steps used to solve the puzzle in addition to speed. To advance in difficulty and achieve higher scores, the user must respond quickly and make as few rotations as possible to fit all the pieces into the presented puzzle.

Memory Lane (Memory)

Memory Lane is a stylized version of the dual n-back task. The user passes by successive apartment buildings. As they pass by each, a person appears in one of its multiple windows and speaks a letter (presented auditorily). Users must remember where the person appeared (which window) and which letter was spoken a given number of buildings back. Users compare these to those for the current buildings, indicating a location match by pressing the left arrow, a spoken letter match by pressing the right arrow, a match on both by pressing both arrows, and withholding a press to indicate a lack of match. The game challenges memory further by asking users to recall all the letters they heard during the round.

Memory Match (Memory)

Memory Match is a 2-back visual working memory task. Users indicate whether the current symbol matches the one presented two symbols previously. A symbol is presented in a box on the right of the screen. The user indicates whether there is a 2-back match, and then the symbol shifts one position to the left. After the next response, the first symbol slides one more position to the left and is now the to-be-matched target. Initially, the current symbol and the previous two are all visible. After the user starts responding correctly, only the current symbol is visible. Game score is calculated from speed and accuracy.

Memory Match Overdrive (Memory)

Memory Match Overdrive is similar to Memory Match (see above) but provides additional challenge because it is a 3-back task. Users must indicate whether the current symbol matches the one presented 3 symbols previously. Similar to Memory Match, all symbols are initially visible but as the user starts responding correctly, only the current symbol remains visible. Game score is calculated from speed and accuracy.

Memory Matrix (Memory)

Memory Matrix exercises short-term memory. In this game, users exercise their spatial short-term memory by remembering the location of squares on a grid. The target squares appear briefly and simultaneously at the beginning of trial. The user must remember the location of all the target squares and click on them after they vanish. Initially, three squares appear and the grid size is 3×3. However, with each correct response, the number of targets is increased by one and the grid size grows. Each time an incorrect response is made, the number of targets is reduced by one and the grid size shrinks. Game score is based on accuracy and level (level increases as the number of target squares increases).

Memory Serves (Memory)

Memory Serves exercises working memory. Playing involves accurately storing incoming information in working memory and continuously updating it as new information comes in. Users assume the role of a bellhop riding up and down in an elevator. On each floor, the bellhop needs to pickup or deliver different types of luggage. The user must keep track of the bellhop's current luggage inventory and only deliver the luggage if it is in the inventory. As the user responds accurately, the bellhop receives more luggage of varying types and elevator stops become briefer, requiring the user to respond more quickly to luggage requests from guests. The game is completed successfully by remembering and dropping off all the correct items to the guests within the time limit. Games last one to three minutes, depending on the difficulty level. Both game score and level are based on the difficulty of the individual deliveries, which depend on the amount and type of luggage to be delivered as well as duration of the elevator stop.

Moneycomb (Memory)

Moneycomb exercises visuospatial working memory. Users must remember the locations of coins hidden within a hexagonal grid (honeycomb) structure. Copper, silver, and gold coins labeled with values 1, 2, and 3, respectively appear in sequence. Users must recall the locations of these coins in the order of their value. Difficulty increases on subsequent trials by increasing the number of coins.

Monster Garden (Memory)

Monster Garden exercises visuospatial working memory. The game challenges users to remember the location of several obstacles that appear only momentarily while navigating the game environment. The game environment is a garden in which obstacles, initially in the form of cartoon monsters, appear briefly one by one. The user must navigate the game environment while avoiding the hidden obstacles to reach a flower. If the user clicks on a space containing an obstacle, a life is lost. The game ends after 3 lives are lost. Levels increase in difficulty by expanding the garden and increasing the number of obstacles that the user must maintain in working memory.

Multiplication Storm (Math)

Multiplication Storm is similar to Raindrops (see below) but restricted to multiplication problems.

Name Tag (Memory)

Name Tag exercises working memory. In this game, users must discover and remember matching pairs of cards within a grid. At the beginning of each session, name tags are displayed that include names paired with the corresponding faces. During game play, users must match name cards with their corresponding face cards.

Observation Tower (Attention)

Observation Tower exercises short-term memory and integration of information across the visual field. This game challenges users to process briefly flashed information quickly. In the game, a set of numbers is presented for a short time and then disappears, leaving only indicators of the numbers' locations. Users must indicate the order of digits they saw in ascending order. Each digit observed and correctly ordered goes towards constructing a tower. The goal is to recall and correctly order as many digits as possible. Observation Tower adapts to user performance by decreasing presentation time as well as increasing the number of digits to remember as the user demonstrates proficiency. Game score is based on accuracy and difficultly level.

Organic Order (Problem Solving)

Organic Order exercises logical and spatial reasoning. This game requires the user to organize items sequentially based on given rules. On each trial, the user is asked to place a number of different fruits and vegetables into a specific order according to a set of provided rules. On some trials, some items are already placed in specific locations and cannot be moved. Periodically, a set of rules will create a logically impossible ordering for the fruits and vegetables. In these cases, the correct response is to select an 'Impossible' button. Correct answers are met with a check mark and the "correct" sound. Wrong answers receive a red X, the "wrong" sound, and one chance to resubmit. A game consists of 7 trials plus 1 additional challenge trial if all previous trials were answered correctly. As users respond correctly, difficultly increases through more complex rules. A higher score is achieved by reaching more difficult puzzles.

Penguin Pursuit (Speed of Processing)

Penguin Pursuit exercises response inhibition, task switching, and spatial reasoning. This game requires users to guide a penguin through a maze while racing against a computer-controlled competitor penguin. The maze repeatedly rotates its orientation, so that users must mentally recode directions – for example, up becomes left. The user must continue to guide the penguin according to the original directions as quickly as possible. If the competitor penguin reaches the end of the maze first, a life is lost. The game ends after 3 lives are used. The task increases in difficulty by increasing rotation frequency, increasing maze size, and increasing speed of the competitor penguin.

Pet Detective (Problem Solving)

Pet Detective exercises planning and spatial reasoning. In this game, users must pick up pets and return them to their homes within a connected map. Users are limited in the distances they can travel and number of pets they can move simultaneously, and thus must plan a route several steps ahead of time to successfully complete the task. Difficulty increases through the number of pets, distance between homes, and map size. Game score is based on efficiency, speed, and number of pets delivered to their respective homes.

Pinball Recall (Memory)

Pinball Recall exercises spatial working memory. This game requires the user to remember the location of multiple objects and predict a ball's path accounting for those objects. The game presents the user with a grid and one or more bumpers that have a specific orientation (±45°). The bumpers disappear after a brief presentation time, after which a point on the outside of the grid is highlighted to designate

the ball's starting location. The user must then determine the path a ball will travel after emanating from the highlighted point, taking the various bumpers into account (balls ricochet at 90° off bumpers), and indicate which point on the outside of the grid the ball will emerge. Difficulty increases with larger grids and more bumpers. Game score is based on accuracy and difficulty level.

Pirate Passage (Problem Solving)

Pirate Passage exercises planning and spatial reasoning. In this game, the user is asked to draw a path for their ship to a treasure chest marked with an X. The user selects the path one square at a time, while avoiding collisions with other ships moving on their own individual paths, marked by color-coded lines. An attempt fails if the user's ship bumps into another ship and succeeds if they reach the treasure chest. Game score and level increase when fewer moves (squares traversed) are made by the user to reach the treasure chest.

Playing Koi (Attention)

Playing Koi exercises visual divided attention and multiple object monitoring. The goal of the game is to feed all the koi. All the koi fish look exactly the same and swim around the pond. The user must feed each exactly once, which challenges the user to focus on multiple targets simultaneously and follow them throughout their journey across the pond. Lily pads may temporarily obscure koi as they travel. Focus is critical to successfully keep track of all the koi and avoid the distractions of other fish moving around the pond. As performance improves, the task becomes significantly more difficult with more fish. Game score is based on accuracy and difficulty level.

Raindrops (Math)

Raindrops exercises basic arithmetic skills and divided attention in a speeded manner. Arithmetic problems (enclosed in water droplets) fall from the top of the screen, and users must enter the answers before the droplets reach the water level at the bottom of the screen. As the user progresses the difficulty increases, with more complex problems falling more frequently. If a drop hits the water, the water level rises, reducing the amount of time a user has to respond to future drops. The game progresses from simple single-digit addition to multiple digit addition, subtraction, multiplication, and division. Game score is related to total number of problems correctly solved.

Rhyme Workout (Memory)

Rhyme Workout is an n-back task that is similar to Memory Match (see above). Rather than matching exact objects, however, cards with words on them appear, and users must decide whether the word n trials ago rhymes with the current word. Thus, the task engages phonemic processing while simultaneously challenging working memory. Users can progress from 1-back to 2-back to 3-back. Game score is based on speed and accuracy of responses in a limited time period.

Rhythm Revolution (Attention)

Rhythm Revolution exercises temporal attention. Users are presented with a record around which note cues are arranged in a rhythmic fashion. The record rotates, and the user listens to the rhythm for two revolutions. Following this, the user repeats the rhythm with the spacebar for four revolutions. As the user graduates to harder trials, eighth notes, sixteenth notes, triples, and fading notes are incorporated.

River Ranger (Speed of Processing)

River Ranger exercises short-term memory and divided attention. This game requires the user to direct attention to multiple stimuli while remembering which specific stimulus was seen in the previous trial. Users must quickly observe simultaneous stimuli and remember their salient visual properties. On each trial, several animals simultaneously emerge from a river for a brief duration. Users must select which one of the animals had appeared during the previous trial. The goal is to make as many correct responses as possible in a fixed period of time. Each game runs for 1 minute and 30 seconds.

Robot Factory (Flexibility)

Robot Factory exercises response inhibition. In this game, users must respond to the temporary appearance of robot parts on three pedestals and select parts to help complete a robot. Some parts that appear will not fit on the robot, in which case, the pedestal indicates an improper part. Users must then withhold their response and not select that pedestal until the part disappears. The game adapts in real time and, as users progress, parts are introduced more quickly and the frequency of improper parts increases. Game score is tied to speed and accuracy.

Rotation Matrix (Memory)

Rotation Matrix exercises visual short-term memory and mental rotation. This game is similar to Memory Matrix (see above), with the addition of a rotation component that makes the task more challenging. In Rotation Matrix, users are asked to remember the location of squares on a grid. The target squares are presented simultaneously for a brief period of time, and after they disappear, the entire matrix rotates 90 degrees left or right. The user must indicate the location of the target squares in their new locations, which requires them to visualize how the target squares would have moved during the rotation. The task begins with a 3×3 grid and one target square for the user to remember. The grid size and number of target squares increase with correct performance and decrease with incorrect performance. Game score is based on accuracy and difficulty level.

Route to Sprout (Flexibility)

Route to Sprout exercises planning through a series of puzzles where users must move an object (a seed) to the goal. Waypoints are connected by paths, but many waypoints contain obstacles (ladybugs). The user can move objects from waypoint to waypoint, but each waypoint can only hold one object at a time. On each trial, users must move the objects one at a time in order to get their seed to the goal, where it is planted. The goal is to complete the puzzle in the smallest number of moves, as quickly as possible. Route to Sprout increases in difficulty by increasing path complexity

Skyrise (Attention)

Skyrise is similar to Observation Tower (see above) and exercises short-term memory and integration of information across the visual field. This game challenges users to quickly process briefly flashed information. On each trial, a series of numbers is flashed briefly on a collection of blocks. Users must remember the numbers and click on the blocks on which they appeared in numerical order from lowest to highest. The goal is to correctly identify as many numbers as possible. Blocks with successfully remembered numbers contribute to building a tower. The goal is for users try to build the tallest tower. Each game consists of 10 trials, and users progress through difficulty levels by correctly identifying all the numbers presented on a given trial. Difficulty is adjusted within each game and is determined by the number of flashed numbers, their spatial configuration, and visual angle subtended. Game score is based on accuracy and corresponding tower height.

Space Junk (Attention)

Space Junk exercises numerical estimation. It is a subitizing task where users are briefly presented with a number of scattered space-themed silhouettes moving on a black background. The silhouettes disappear and users must indicate how many objects they saw. In this task, the user generally does not have enough time to count the objects on the screen and must instead estimate how many there were. Difficulty is increased by increasing the number of objects and amount of motion.

Space Trace (Flexibility)

Space Trace exercises spatial short-term memory and the generation of new patterns. The goal of this game is to generate as many unique visual patterns as possible. The user is asked to draw 5 lines through a series of presented dots (dots can be used more than once in a pattern). Once 5 lines have been drawn, the design is judged as either unique or repeated, depending on whether the user has drawn this same design this game. Each unique design gains the user points and receives positive feedback. If the design is not unique, the user receives feedback that it is a repeat. The design is then cleared and the user may begin another. The game lasts for 45 seconds, plus bonus time earned for correct designs. Game score is based on the number of unique designs drawn during the time period.

Spatial Speed Match (Speed of Processing)

Spatial Speed Match exercises short-term memory. This game is similar to Speed Match (see below), except that the stimuli on the cards are three circles arranged in a triangle with one circle filled, rather than the shapes used in Speed Match. This challenges spatial discrimination rather than object discrimination.

Speed Match (Speed of Processing)

Speed Match exercises short-term memory. In this game, a stack of cards with shapes on them is flipped one at a time, and the user indicates whether the current card matches the previous card (1-back). Performance is measured by how fast and accurate a user can perform in a fixed timeframe, and the experience is designed such that successful users will increase speed across sessions while maintaining high accuracy.

Speed Match Overdrive (Speed of Processing)

Speed Match Overdrive is an advanced version of Speed Match and exercises working memory. In Speed Match Overdrive, symbols have shape and color attributes, and the user indicates whether the current symbol matches the previous symbol exactly, partially, or does not match. This task challenges the user to respond quickly and accurately. The response is biased toward accuracy by including increasing bonuses for increasingly long chains of correct responses. Game score is based on speed and accuracy.

Speed Pack (Speed of Processing)

Speed Pack exercises spatial reasoning and visualization skills in a physically intuitive task. Users are presented with suitcases filled with objects. One of these objects (a camera) is moveable, and users must position that object such that when the suitcase is folded, none of the objects overlap each other. Trial complexity is adapted based on performance – more objects and more folds are introduced to users

completing trials quickly and accurately. Users are encouraged to pack as many suitcases as possible within a limited amount of time. Game score is based on speed and accuracy.

Splitting Seeds (Speed of Processing)

Splitting Seeds exercises counting and numerical estimation. This game requires using a stick to divide a collection of seeds into two equal parts. On each trial, an even number of seeds and a stick appear on the screen. The seeds cannot be manipulated, so the user must rotate the stick on the board to divide the seeds into two equal parts. Users try to quickly and accurately divide seeds on as many trials as they can within the game duration of 1 minute and 30 seconds. Difficulty is adjusted by increasing the number of seeds presented. Both difficulty level and score are based on speed and accuracy.

Star Search (Attention)

Star Search exercises selective attention. In this game, users must find a single unique object among a field of objects. The objects are shapes with primitive physical characteristics, such as color, orientation, motion, and shading. Sets of other objects share characteristics with the target object to distract users. If users find the unique object quickly, the game increases in difficulty with the introduction of characteristics that are harder to tell apart. For example, the game begins with different shapes with different colors. At higher levels, the shapes and colors are identical, but the target object is rotated in a different direction from that of the distractor objects. The number of objects that share some, but not all, characteristics also increases. Game score is based on speed and accuracy.

Subtraction Storm (Math)

Subtraction Storm is similar to Raindrops (see above) but restricted to subtraction problems.

Taking Root (Language)

Taking Root exercises vocabulary knowledge and verbal fluency. The game features two phases of six trials each, a root definition learning phase and a word composition phase. On each trial of the first phase, a root is given along with three words that include the root. The words can be tapped on to give a definition. On each trial of the second phase, a partial word is given with component roots to fill in. The roots come from a selection of roots learned previously. Level (difficulty) is adjusted based on speed and accuracy. Points are awarded on each trial based on accuracy, speed, and difficulty.

Tidal Treasures (Memory)

Tidal Treasures exercises short-term memory and working memory. In this game, an increasing number of objects wash up on a beach, and in each trial the user must select an object not previously selected. Items include things like seashells, kelp, flotsam, and jetsam that one might expect to find on a beach. More items appear in each trial, taxing the user's memory, until the user selects an object that had been previously selected. As users progress through rounds, difficulty also increases by decreasing the uniqueness and distinctness of objects. The task differs from other working memory exercises by allowing users to choose the objects they wish to remember, which engages other executive functions. Game score is based on the number of unique objects identified.

Top Chimp (Attention)

Top Chimp exercises short-term memory and integration of information across the visual field. In this game, the user briefly sees scattered poker-style chips with numbers on them. The numbers disappear

and the user must order the chips in ascending order. Users compete against computer opponents and gauge their own ability by betting different numbers of chips depending on their confidence in beating the opponent. Difficulty is increased by decreasing presentation time and increasing the number of chips numbers to be remembered, as well as increasing the number of chips necessary to bet in order to beat the computer opponent.

Top That (Math)

Top That exercises fast numerical calculation and estimation. In this game, users are asked to quickly calculate and/or accurately estimate prices in order to swap/trade for ever-increasing valued prizes before time runs out. The goal for the user is to end their session with a much higher priced prize than what they started with. As the user trades for higher and higher tiered offers, the game introduces challenges that make the price calculation more difficult to compute in the time given. These challenges include multipliers, addition/subtraction, and percentages. All offers are available only for a limited time, expire, and are replaced quickly. Timing of the offers is staggered. This keeps the pace of the game quick and encourages the player to estimate rather than calculate. The game lasts for 50 seconds. Game score is based on accuracy (selecting a top offer) as well as overall ending offer value and level reached.

Train of Thought (Attention)

Train of Thought exercises divided attention. This game requires users to a direct a set of color-coded trains to their destinations. Trains emerge from a tunnel and travel continuously along a branching set of tracks. Users must click switches at the branch points in order to correctly direct the trains to their destinations. Train frequency changes within a session to match a user's performance, increasing with correct deliveries and decreasing with incorrect deliveries. When users perform well, delivering nearly all trains to their correct destination, the user advances in level and the number of stations increases. Score is based on level and number of trains directed to the correct station.

Trouble Brewing (Attention)

Trouble Brewing exercises divided attention. In this game, users manage a coffee shop. Coffee orders are introduced and must be filled by adding the requested ingredients to a cup and then starting the coffee dispenser. Care must be taken to stop the machines at the right time, so that the cups are filled but not overfilled. Users must plan out their strategy, using multiple machines to make use of their time most efficiently, and to serve all customers as quickly as possible. At higher levels, users must manage multiple screens, taking orders on one and executing them on two others. Game score is based on speed and accuracy.

Word Bubbles (Language)

Word Bubbles exercises verbal fluency. A three-letter word stem is presented and users are given 1 minute to enter as many words as possible that begin with that stem. To receive high scores, users must enter multiple words of various lengths ranging from 4 letters to 13+ letters.

Word Bubbles Rising (Language)

Word Bubbles Rising enhances the gameplay of Word Bubbles (see above) by introducing 4 and 5 letter word stems in subsequent rounds, which are shortened to 3 letters after a set time interval. Game score is based on the number of words generated, length of words, and difficulty level.

Word Snatchers (Language)

Word Snatchers exercises vocabulary and verbal fluency. This game involves solving anagrams. On each of 12 trials, the user is presented with the definition of an unknown word and a set of letters. The letters begin to rise, indicating a set time limit to solve the anagram, as the user can no longer guess once the letters reach the top of the screen. The user may request hints but doing so lowers their overall score. The number of letters in the word range from 3 to 10. Difficulty of the word, based on its length and frequency in English, is adjusted trial to trial based on the user's performance. Game score is based on accuracy, speed, and difficulty.

Word Sort (Problem Solving)

Word Sort is similar to By the Rules (see above). However, instead of pictorial items, words must be classified. Word categories can include the number of letters, typeface, starting and ending letter, and semantic relationships.
