# Supplementary material for: Transfer of learning: Analysis of dose-response functions from a large-scale, online, cognitive training dataset
Source: PLoS One. 2023 May 17;18(5):e0281095. doi: 10.1371/journal.pone.0281095 (PMC10191334; doi:10.1371/journal.pone.0281095)
Supplement: S1 File — (PDF) [file pone.0281095.s003.pdf]

S1 Additional analyses. D-R function of effect sizes for NCPT Grand Index

D-R functions showing the relation between amount of CT and effect size may be useful for planning efficacy studies. Efficacy studies typically compare change scores on an assessment between treatment and control groups. Although they don't involve a control group, D-R functions can provide an analogous comparison. In the analysis reported here, change scores for a control group were approximated by those of participants (N = 6,765) who engaged in 25 or fewer games between the two assessments. The remaining participants (N = 100,240) were divided into deciles based on their number of gameplays between the two assessments. Each of these 10 groups (N = 10,024) corresponds to a treatment group with a different dose of CT. Effect sizes (Hedges' g) for their change scores on the NCPT Grand Index, relative to those for the "control group," are shown in the figure below.

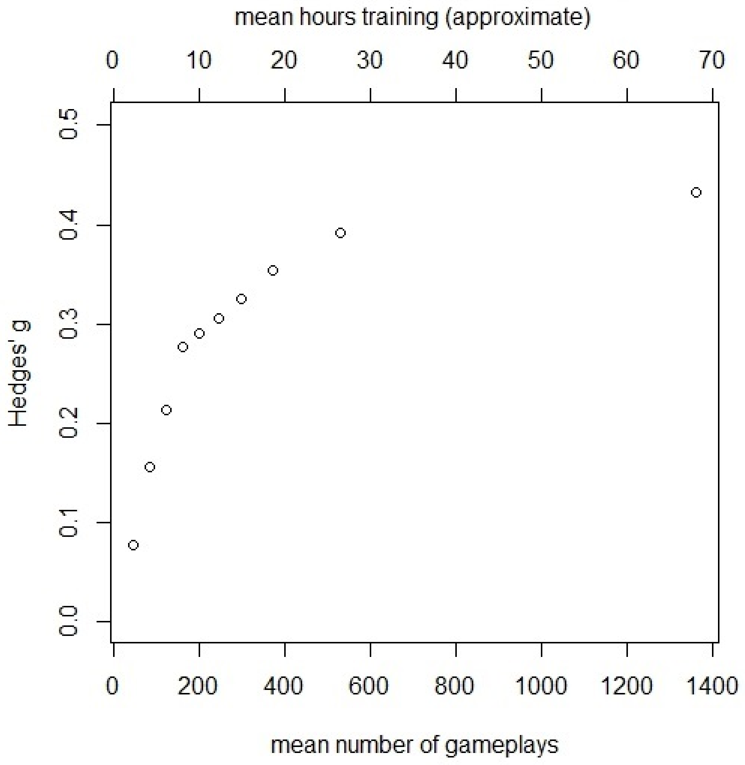

Fig A. Dose-response function relating CT dose and effect size. Dose is shown in terms of mean number of games (bottom x-axis) and mean CT duration based on 3 minutes per gameplay (top x-axis). Effect size is Hedge's g for change score on the NCPT Grand Index. Each dot corresponds to a decile of participants based on their amount of CT. The effect size for each decile was calculated by comparing their change scores to those of a group with minimal CT (25 games or less).

The figure may help to provide an initial estimate of how much CT would be required in an efficacy study to reach various effect sizes. To make the D-R function more applicable to CT programs other than Lumosity, dose is expressed both in terms of the mean number of Lumosity gameplays (bottom x-axis) and mean duration of CT based on 3 minutes per gameplay (top x-axis). As can be seen, effect size increases with dose, but with diminishing returns. For example, a moderate amount of CT (300 games, 15 hours) led to an effect size of about 0.325. Doubling the amount of CT increased the effect size only to about 0.4. Note that these effect sizes may be slight underestimates of what would be observed in an efficacy study. One reason is that the "control" group here actually did receive some CT (albeit 25 games or less). Also. participants in each of the 10 treatment groups varied in their exact amount of training (and game choices). This would be expected to increase the variance of change scores in each group, and hence diminish effect size.
