# Supplementary material for: Transfer of learning: Analysis of dose-response functions from a large-scale, online, cognitive training dataset
Source: PLoS One. 2023 May 17;18(5):e0281095. doi: 10.1371/journal.pone.0281095 (PMC10191334; doi:10.1371/journal.pone.0281095)
Supplement: S2 File — (PDF) [file pone.0281095.s004.pdf]

S2 Additional analyses. Age as a continuous variable

A major finding of the current study is that the effects of repeated testing on performance of the NCPT diminished with age, while those of transfer from CT remained the same. One question that might be asked about this finding is whether it depends on the specific categories into which the age variable was divided, i.e., on the number of and boundaries between age bins. The three ANCOVAs presented below each correspond to an analysis in the main body of the paper and address this question by showing that a similar pattern of results occurs when age is treated as a continuous variable.

Additive effects of age and amount of CT

The first ANCOVA corresponds to an analysis comparing the parameters of four exponential functions fit to data from participants in four different age categories (Table 3). Because this approach required discrete age categories, a different approach was adopted in order to treat age as a continuous variable. Besides age, the ANCOVA includes log1p(number of games played between NCPTs) as an independent variable. As in the preliminary analyses (Fig 3, Table 2), a log function was selected because it can be included as a variable in a linear model and captures the diminishing returns of transfer with increasing gameplay (Fig 5). An interaction between age and this variable in their effects on the change in NCPT performance would indicate that transfer varies with age. Alternatively, an additive relation between the two variables would indicate that only the effects of repeated testing vary with age. The results of the ANCOVA are shown in Table A.

Table A. Results of ANCOVA examining the combined effects of age as a continuous variable and the number of games between NCPT assessments on change in the NCPT Grand Index (GI).

Response: Change in GI, Model: Change in GI ~ Age * Log1p(Gameplays), Sum of Squares: Type II				
**Effect**	**Sum of Squares**	**Degrees of Freedom**	**F Value**	**P Value**
**Age**	12,212	1	257.5778	<2e-16 ***
**Log1p(Gameplays)**	65,777	1	1,387.4228	<2e-16 ***
**Age:Log1p(Gameplays)**	1	1	0.0185	0.8918
**Residuals**	4,435,534	93,558		

As can be seen in the table, both Age and Log1p(Gameplays) had additive effects on the change in GI. There were significant main effects for both but no interaction. The effects of transfer on change in GI remained constant across age. Likewise, while change in GI varied with age, this change did not depend on the amount of gameplay, i.e., was due to repeated testing on the NCPT.

Age effects on features of empirical D-R functions

The primary approach adopted in this study involved using the intercept and asymptote-intercept of fit exponential functions to measure respectively the effects of repeated testing and transfer. To provide converging evidence on how these effects varied across age, we also examined how features of the empirical D-R function that approximate these exponential parameters varied with age. The intercept was approximated by change in GI for participants with little or no gameplay between NCPTs (< 26

games, N = 5,827), while the asymptote-intercept was approximated by the difference in the amount of change in GI between participants with little or no training and those with asymptotic levels of gameplay (> 999 games, Fig 5, N = 4,674) between NCPTs. The remaining two ANCOVAs correspond to these analyses but replace the categorical age variable with a continuous one. Their results are presented in Tables B and C.

Table B. Results of ANCOVA examining the effects of age as a continuous variable on the empirical approximation of the D-R function intercept (change in GI for participants with < 26 gameplays between NCPT assessments).

Response: Change in GI, Model: Change in GI ~ Age, Sum of Squares: Type II				
**Effect**	**Sum of Squares**	**Degrees of Freedom**	**F Value**	**P Value**
**Age**	1,324	1	24.953	6.047e-07 ***
**Residuals**	309,052	5,825		

Table C. Results of ANCOVA examining the effects of age as a continuous variable on the empirical approximation of the D-R function asymptote-intercept (change in GI for participants with > 999 gameplays between NCPT assessments minus change in GI for participants with < 26 gameplays between NCPT assessments).

Response: Change in GI, Model: Change in GI ~ Age * Empirical_A-I, Sum of Squares: Type II				
**Effect**	**Sum of Squares**	**Degrees of Freedom**	**F Value**	**P Value**
**Age**	1,937	1	37.2987	1.049e-09 ***
**Empirical_A-I**	31,027	1	597.5593	< 2.2e-16 ***
**Age:Empirical_A-I**	6	1	0.1138	0.7359
**Residuals**	545,031	10,497		

The main effect of Age in Table B on Change in GI corresponds to an effect on the intercept of the D-R function. The main effect of Empirical_A-I in Table C on Change in GI corresponds to the maximum effect of transfer, and the lack a significant interaction between this variable and Age indicates that the maximum effect of transfer did not vary with age.

Conclusions

The three ANCOVAs presented here support the same conclusions as their corresponding analyses in the main body of the paper. The effects of repeated testing on the NCPT diminished with age, while those of transfer from CT remained constant. Thus, these conclusions do not depend on the particular age categories adopted in the main analyses of this study.
