# Supplementary material for: Transfer of learning: Analysis of dose-response functions from a large-scale, online, cognitive training dataset
Source: PLoS One. 2023 May 17;18(5):e0281095. doi: 10.1371/journal.pone.0281095 (PMC10191334; doi:10.1371/journal.pone.0281095)
Supplement: Attachment — Submitted filename: Review PLOS Luminosity.pdf [file pone.0281095.s005.pdf]

Dear Allen,

Overall I really enjoyed reading this paper – the methods were well done and of high quality yet they need to be clarified to make this manuscript suitable for publication. In the future, it would be good to see something preregistered or with a held-out test set. The data is openly accessible which is great, while I think code should be included it's not a requirement.

1. The intro is well written and I have no comments except I think the use of CT & CCT is unnecessary (along with GI, which after being introduced could just be called 'improvement or change'; and cognitive training can just be written as training after you introduce it) yet this is the author's stylistic choice and is a mere suggestion.

The main section that I think needs to be re-written for clarity is the results section; this may also be due to the methods section lacking the relevant info. I have read it a few times and it is still unclear this can be partially fixed by making the tables and figures clearer – yet exactly what is happening needs to be explicitly explained. Simply things like reiterating that you are fitting a DR model to each subgroup at each section on the results (we fit X models per subgroup).

Specific points:

2. Plots still need to be in higher resolution
3. Plots please also change the unadjusted significance to an (x) or another symbol other than a star, as an alpha of .05 should correspond to a single *.
4. Plots 6, 9 & 10 are way too close to box plots with IQR, please change them to standard coefficient plots (you can revert the axis if you want the vertical). There are also dots on them and they are missing parts – yet if you make new coefficient plots this doesn't matter.
5. I would like standardized effects (mean = 0, sd = 1) throughout, the authors don't make a strong claim about intelligence and IQ point so why scale by them... It's much clearer to a wide readership to be in standard units. Or at least standard units next to IQ points (for example line 298).
6. Amount of gameplay (line 273) this distribution is very wide and low… Why do you have subjects that never played games…? This can't possibly be a normal population; it would be good to see a robustness check with meaningful ranges. Considering those that did no games as test-retest neglects they're not normal subjects. Unless it is a non-contact control? But it doesn't seem this is the case…

these are weird subjects that signed up for Lumiostiy and did no luminosity and came back weeks later for t2???

7. Clarify that you are making age brackets because it's a modeling limitation? The brackets you choose make sense to me and I have no criticism about that but it will clarify to the reader why you went from a continuous measurement. Also, include counts for these brackets in table 1!!! The mean & SD of age are unnecessary since you don't use them yet the graph is super nice.
8. Time is related to the number of games. There is obviously some error in when subjects took the 2nd test (some doing it later than others). Show this & maybe prove it is independent (or small) of demographics or add it as a covariate. Have you thought about this? Maybe I missed it?
9. <u>This is my major point</u>: The results section corresponding to tables 3 & 4: It doesn't help that you have table 2 which follows a standard table reporting format of linear models. These tables give a false impression everything is in a single model when in reality each line (row) corresponds to a single model – it doesn't help for clarity that you subtract from the first model.
    a. This section and these tables need to become much clearer; spell out exactly what you are showing. (e.g., we fit # number of models, then we wanted to test the differences of X model from X model; The model for high school females ages 14- 40…)
    b. One way to make the tables clearer is not to subtract model 1 (i.e., the reference) from the other.
    c. It is unclear the statistical tests happening here, is it each model's coefficient from zero or is it the contract of betas between models? Some of your claims are on contrasts so if you would like to keep them you need to do a contrast test (preferably a full set; or at least the contracts for your claims age 1 vs age 2; age 2 vs age 3; age 3 vs age 4 etc…). If this isn't the case the way the tables are made is suuuuper misleading. That is when a model fit on young people has a beta of .1 a statistical test is needed to determine if .08 in old people is significantly different. I couldn't find this information clearly spelled out.
    d. Honestly, for the tables, I would literally write model 1; model 2; model 3 on each row and stop subtracting the reference model. I just realized you have an empty column where you could write "Model" above everything (and then get rid of the confusing reference-based subtraction; depending on how the statistical tests are done; point c).

e. Another clear way to show they are separate models is to write the number of subjects fit in each as a row of the table.
f. It took me a while to figure out why the only age differences model (line 374) exists… I think this is due to the model framework used (i.e., necessitating small groups of covariates being fit)? What I am not a fan of is claiming because you didn’t an effect in the model before, these demographics – which you show at the start of the results to be related to gameplay – are unnecessary. This is not how covariate control works or should be used; a non sig covariate is still potentially doing something and it should be there based on causal a priori hypotheses (Wysocki, Lawson, Rhemtulla, 2022, Adv. in Methods and pract. in psyc. sci.). I sympathize with your justification for an ‘increase of power’ because you are fitting models to the unity of groups (e.g., 18-40, HS, F) yet it's not an adequate reason to ignore covariates. If you would like to keep it a reason please write and point out your smallest group as an example. It’s okay to write that you have modeling constraints and that you just can’t do it/are limited by DR functions (is this the main reason?). If you really, really want to focus on this analysis another way to proceed is to residualize the change score for those left-out demographics. Personally, I think this section should be put in the SI and referenced in a sentence in the analysis before. How you proceed with it is totally your choice – yet the justifications are what I have issues with.
g. I think lines 387 - 389 are a mistake, the table doesn’t seem to reflect this and has similar coefficients to Table 3?

The discussion is well written and I have very little to comment on except the following:

10. Lines 623-632 could be entirely trimmed.
11. Line 633 has a very good point and should definitely stay.
12. I have issues with the limitation section, I feel like with this type of study we should just own the limitation and not try to skirt around it or justify it. Major issue with lines 657-659; Grammatifal reasoning didn’t fit… you don’t even include it in the table… it can’t be used ‘when convenient’. If you have a really good reason you think this isn’t a limitation then spell it out, yet the current justifications don’t pass muster, and it's okay, every study has limitations that don’t need to be explained away.

Overall a very cool study and well done. I think by making the results section clearer this will turn out to be a great paper. It would be cool to see future research at Luminosity minimizing confounders by altering the number of prompts/notifications or changes in the app followed by a regression-discontinuity design.

In the interest of transparency, I sign all my reviews the comments made are meant to be constructive and I think that this paper has great promise .

Nicholas Judd, Ph.D.
