# Supplementary material for: Transfer of learning: Analysis of dose-response functions from a large-scale, online, cognitive training dataset
Source: PLoS One. 2023 May 17;18(5):e0281095. doi: 10.1371/journal.pone.0281095 (PMC10191334; doi:10.1371/journal.pone.0281095)
Supplement: Attachment — Submitted filename: Responses to reviewers and Journal.docx [file pone.0281095.s006.docx]

**Reviewer 1**

I appreciate the opportunity to review this article. Being immersed in this literature with Lampit et al., I read this with much curiosity. Comments and suggestions are below.

1. Overall, this is well written, but not necessarily easy to follow. This is very dense material which is not necessarily a bad thing, but being someone who is very familiar with the literature, it was difficult to understand.

We have responded to a number of your comments below (3, 5, 6, 7, 9, 11) in ways that we think will make the paper somewhat easier to follow. Thanks especially for comment 6, which helped us make the paper meaningful to a larger audience.

2. Methodologically, this is a strong article with a large sample size.

Thanks. We think that the large sample size is one of the study’s greatest strengths.

3. I am not sure I understand what DR functions are after reading this article (lines 58-60). I guess it has something to do with background characteristics and such. I was never quite sure what this meant. To me, this is simply looking at covariates of dose-response.

The concept of a dose-response (D-R) function is central to the paper. After reading your comment, we realized that we never provided an explicit definition. One can now be found on line 63.

4. The review and integration of the literature is on point.

Thanks. We are encouraged by your feedback.

5. I think GI stand for Grant Index (line 229). I figured this out later. So I assume this is the just the overall global cognitive composite.

That’s correct. It’s important that we be clear about this, since GI refers to a major dependent variable of the study. After reading your comment, we realized that, when we defined the Grand Index (on what was formerly line 229), we never indicated that its abbreviation was GI. This has been rectified (line 322). We also provide a reminder on line 482 that GI is a measure of overall performance on the NCPT.

6. I was glad to see Figure 5 which shows that after a while, there is diminishing return on the therapeutic benefits of these games; that parallels Lampit et al.’s work. It is not clear to me how much that translates into time, it is only express in games. As someone who works with these games, I need to know TIME for this to be useful.

We agree that knowing dose in terms of the amount of time spent training is important for clinical research and practice. Since, a single game lasts on average about 3 minutes, the cumulative number of hours spent training is approximately equal to the number of games played divided by 20. This is now stated where the to-be-interpreted features of the D-R plot are introduced (line 481).

People in clinical fields might also be interested in S1 Additional Analyses, where effect size (Hedge’s g) is plotted as a function of training (both number of games and time).

7. I had trouble interpreting Figure 3, but the other figures made sense (more or less)

To help make Fig. 3 more interpretable, the figure caption has been revised somewhat (lines 445-448).

8. A strength of this study is that they examined the impacts of training on various cognitive domains (Table 5 and Figure 8).

Agreed.

What is not there is what type of training is provided; it seems to be all lumped together (and I get it…there is only so much you can do with the data).

We agree and elaborate on this point in the Limitations section (lines 1035-1044).

“Other limitations stem from the broad range of CT, which involved a large number of different games and targeted multiple cognitive domains. Participants were free to choose which games to engage in, and daily suggestions were offered to encourage diversity (see Methods). While such cross-training may be an effective form of CT, it made it difficult to study the effects of training in specific cognitive domains. Nonetheless, future analyses of this dataset could classify participants with respect to the relative amounts of training they received in each of several cognitive domains. As mentioned, the games can be organized on the basis of their primary cognitive demands (Memory, Attention, Flexibility, Problem Solving, Speed of Processing, Math Skills , or Language Skills).  Alternatively, each game can be characterized by its loadings on a set of latent cognitive factors [41].  A more direct approach, involving other studies, would be to limit CT to a specific cognitive domain. …”

9. Some of the figures were blurry and hard to read. I just really gave up and tried to glean what I could from the text.

We apologize for this. We had failed to detect that resolution in some of the figures was reduced during submission. The figures in the resubmission are all 300 dpi.

10. Some interesting findings
a. “we did not detect effects of either gender or education on the change in NCPT performance following CCT”

Still, we do accept the possibility (and, in fact, hope) that education can influence the effects of CT (lines 928-932).

b. “While the effects of direct practice on the NCPT diminished with age, those of transfer from CT remained constant”

We consider this to be one of the major findings of the study!

11. Line 633 – Not sure what is meant by “a useful converging operation”.

We have revised the sentence containing this phrase (lines 997-998) to clarify its meaning.

12. In the Limitation section, one could speculate on how personality could also impact the cognitive training outcomes. There has been some recent work on that.

This is definitely an interesting question and one directly related to the important goal of individualizing CT. We are very interested in traits like growth mindset and have informally observed a variety of ways in which individual participants engage with our CT program. But we are not yet able to make public statements, even speculations, on this topic.

13. In the limitations, I am glad to see that “motivation” and “expectation of improvement” is provided. These are important components.

We agree.

14. Overall, I think this article will be seminal, but it is very obtuse and difficult to read because it is very dense. I defer to other reviewers in someone making this more readable if possible.

We hope you’re right about the article’s impact. Thanks for helping us make it more accessible and of interest to a wider audience.

**Reviewer 2**

Overall I really enjoyed reading this paper – the methods were well done and of high quality yet they need to be clarified to make this manuscript suitable for publication. In the future, it would be good to see something preregistered or with a held-out test set. The data is openly accessible which is great, while I think code should be included it’s not a requirement.

1. The intro is well written and I have no comments except I think the use of CT & CCT is unnecessary (along with GI, which after being introduced could just be called ‘improvement or change’; and cognitive training can just be written as training after you introduce it) yet this is the author's stylistic choice and is a mere suggestion.

While we’re going to stick with CT and GI, CCT has been replaced with just CT.

The main section that I think needs to be re-written for clarity is the results section; this may also be due to the methods section lacking the relevant info. I have read it a few times and it is still unclear this can be partially fixed by making the tables and figures clearer – yet exactly what is happening needs to be explicitly explained. Simply things like reiterating that you are fitting a DR model to each subgroup at each section on the results (we fit X models per subgroup).

You are absolutely right. An important shortcoming of the paper was the lack of clarity about the models used to obtain parameter estimates for the exponential functions fit to the data. While these models have not changed, they are now described in considerable detail (lines 331 – 411) in a new section in the Methods. It is also made clear in both the Methods and Results which models were applied to which analyses.

We think that, by making clear the actual models employed, these descriptions address most of your major point (9, a-f). Providing clear detailed descriptions of the models also strengthened the paper. Thanks for your feedback on this!

Specific points:

2. Plots still need to be in higher resolution

Done. All figures are now 300 dpi. We again apologize for some of the original figures being too fuzzy to read.

3. Plots please also change the unadjusted significance to an (x) or another symbol other than a star, as an alpha of .05 should correspond to a single *.

Done. See Figs 9 and 10.

4. Plots 6, 9 & 10 are way too close to box plots with IQR, please change them to standard coefficient plots (you can revert the axis if you want the vertical). There are also dots on them and they are missing parts – yet if you make new coefficient plots this doesn’t matter.

Done. They are now coefficient plots.

5. I would like standardized effects (mean = 0, sd = 1) throughout, the authors don’t make a strong claim about intelligence and IQ point so why scale by them... It's much clearer to a wide readership to be in standard units. Or at least standard units next to IQ points (for example line 298).

We appreciate the comment, and the goal of clarity for a wide readership. However, the units used in this paper follow the convention used for the NeuroCognitive Performance Test, as first described in [Morrison et al. (2015)](https://www.frontiersin.org/articles/10.3389/fpsyg.2015.01652/full?source=post_page---------------------------). Since its introduction the NCPT has been used in large healthy and clinical populations (e.g., as part of the UCSF Brain Health Registry), always with the units used here. Among the studies to use the NCPT, the large RCT conducted by [Hardy et al. (2015)](https://journals.plos.org/plosone/article?id=10.1371/journal.pone.0134467) that is described in this paper also used these units. To maintain consistency with the literature and facilitate a comparison across studies, we believe it is appropriate to keep the units as written.

6. Amount of gameplay (line 273) this distribution is very wide and low… Why do you have subjects that never played games…? This can’t possibly be a normal population; it would be good to see a robustness check with meaningful ranges. Considering those that did no games as test-retest neglects they’re not normal subjects. Unless it is a non-contact control? But it doesn’t seem this is the case… these are weird subjects that signed up for Lumiostiy and did no luminosity and came back weeks later for t2???

As with other program memberships -- both traditional (e.g., gymnasiums) and online (e.g., online courses or app subscriptions) -- there is a wide range of program use, including a number of people who do not use the program for which they signed up. Lumosity is no different, and these are indeed normal participants. An important point is that all participants received an email reminder when they were eligible to take their NCPT test at the second timepoint (T2); therefore, even those participants who had not engaged with their training were notified to return. As you imply, it may have been difficult to expect participants to return for T2 with no notification or reminder.

7. Clarify that you are making age brackets because it’s a modeling limitation? The brackets you choose make sense to me and I have no criticism about that but it will clarify to the reader why you went from a continuous measurement. Also, include counts for these brackets in table 1!!! The mean & SD of age are unnecessary since you don’t use them yet the graph is super nice.

Age brackets are now provided in the text on lines 550-551. The rationale for bracketing age to make it a categorical variable can be seen in the descriptions of Models 2 and 3 (new material, lines 362-411).

8. Time is related to the number of games. There is obviously some error in when subjects took the 2nd test (some doing it later than others). Show this & maybe prove it is independent (or small) of demographics or add it as a covariate. Have you thought about this? Maybe I missed it?

Astute observation. We had indeed considered what effect variability in when participants took the second NCPT might have on the observed D-R functions. Such an effect, if it occurred, would be that our estimates of a positive dose-response relation are in fact overly conservative. This is because longer time intervals between the first and second NCPTs provide, not only the opportunity to play more games in the interim, but also a greater opportunity to lose the benefits of having taken the first NCPT at the time of the second. In actuality, the positive correlation between number of gameplays and days between the two NCPTs was quite small (0.0069). For the above reasons, and because an adequate treatment would be rather lengthy, we had decided previously that a discussion of this issue was unnecessary and would be a distraction from the main narrative.

However, after reading your comment, we felt it important to include in the paper a discussion of this issue in relation to age effects on D-R functions. So, we included a new paragraph to show that the observed effect of age was not an artifact resulting from variability in time between the two NCPTs (lines 715 – 726). Briefly, we explain how the observed decrease in the effects of repeated testing (D-R function intercept) with age could result if there were a positive correlation between age and time interval between the two NCPTs. We then report the actual correlation, which was small and negative (-0.078). So, as with the positive relation between CT dose and amount of training, our estimate of age effects on the D-R functions is, if anything, overly conservative.

9. This is my major point: The results section corresponding to tables 3 & 4: It doesn’t help that you have table 2 which follows a standard table reporting format of linear models. These tables give a false impression everything is in a single model when in reality each line (row) corresponds to a single model – it doesn’t help for clarity that you subtract from the first model.

***Tables 3 and 4 actually do report the results of a single model!*** The values reported in each table were obtained by fitting a single model containing several exponential functions, each governed by dummy variables corresponding to one of the involved demographic levels. For example, all values in Table 4 were estimated simultaneously by fitting a 12-parameter (3 exponential x 4 age levels) model.

The three models employed in this study are described in detail (lines 331-411) in a new section in the Methods. The descriptions include explicit equations for the models and indicate which analyses in the Results each was applied to. The models are also referenced in the Results.

We believe that comments a-f below are all based on the impression that each row in Tables 3 and 4 corresponded to a different model. While this was not the case, it does highlight the importance of providing explicit descriptions of the fit models, which we have now done.

a. This section and these tables need to become much clearer; spell out exactly what you are showing. (e.g., we fit # number of models, then we wanted to test the differences of X model from X model; The model for high school females ages 14- 40…)

b. One way to make the tables clearer is not to subtract model 1 (i.e., the reference) from the other.

c. It is unclear the statistical tests happening here, is it each model's coefficient from zero or is it the contract of betas between models? Some of your claims are on contrasts so if you would like to keep them you need to do a contrast test (preferably a full set; or at least the contracts for your claims age 1 vs age 2; age 2 vs age 3; age 3 vs age 4 etc…). If this isn’t the case the way the tables are made is suuuuper misleading. That is when a model fit on young people has a beta of .1 a statistical test is needed to determine if .08 in old people is significantly different. I couldn’t find this information clearly spelled out.

d. Honestly, for the tables, I would literally write model 1; model 2; model 3 on each row and stop subtracting the reference model. I just realized you have an empty column where you could write “Model” above everything (and then get rid of the confusing reference-based subtraction; depending on how the statistical tests are done; point c).

e. Another clear way to show they are separate models is to write the number of subjects fit in each as a row of the table.

f. It took me a while to figure out why the only age differences model (line 374) exists… I think this is due to the model framework used (i.e., necessitating small groups of covariates being fit)? What I am not a fan of is claiming because you didn’t an effect in the model before, these demographics – which you show at the start of the results to be related to gameplay – are

unnecessary. This is not how covariate control works or should be used; a non sig covariate is still potentially doing something and it should be there based on causal a priori hypotheses (Wysocki, Lawson, Rhemtulla, 2022,

Adv. in Methods and pract. in psyc. sci.). I sympathize with your justification for an ‘increase of power’ because you are fitting models to the unity of groups (e.g., 18-40, HS, F) yet it's not an adequate reason to ignore

covariates. If you would like to keep it a reason please write and point out your smallest group as an example. It’s okay to write that you have modeling constraints and that you just can’t do it/are limited by DR functions (is this the main reason?). If you really, really want to focus on this analysis another way to proceed is to residualize the change score for those left-out demographics. Personally, I think this section should be put in the SI and referenced in a sentence in the analysis before. How you proceed with it is totally your choice – yet the justifications are what I have issues with.

g. I think lines 387 - 389 are a mistake, the table doesn’t seem to reflect this and has similar coefficients to Table 3?

Those lines (plus the preceding one) state “Medians and 95% confidence intervals of the bootstrapped values at each age level are shown in the figure. Here the parameter values for non-reference levels are shown directly, rather than as differences from those at the reference level. As in Tables 3 and 4, the intercept can be seen to decrease with increasing age, while rate and asymptote–intercept remain relatively constant.”

We think you are saying that Table 4 shows parameter values similar to those in Table 3, but different from those in the figure (6). If so, you are correct that the parameter values in Table 4 agree with those for age in Table 3. (Which means the two analyses, each involving a different model, produced similar findings for age.) However, Table 4 and Figure 6 do in fact show the same values. Perhaps the apparent difference is due to how they are represented. In contrast to the two tables, in Figure 6 “the parameter values for non-reference levels are shown directly, rather than as differences from those at the reference level.”

The discussion is well written and I have very little to comment on except the following:

10. Lines 623-632 could be entirely trimmed.

We like this paragraph and want to emphasize the described affordances of D-R functions.

11. Line 633 has a very good point and should definitely stay.

OK.

12. I have issues with the limitation section, I feel like with this type of study we should just own the limitation and not try to skirt around it or justify it. Major issue with lines 657-659; Grammatifal reasoning didn’t fit… you don’t even include it in the table… it can’t be used ‘when convenient’. If you have a really good reason you think this isn’t a limitation then spell it out, yet the current justifications don’t pass muster, and it's okay, every study has limitations that don’t need to be explained away.

While we think that discussing the parsimony of an alternative explanation for our findings is not unreasonable, we agree that our selective consideration of results from the grammatical reasoning subtest is somewhat arbitrary. So, we removed the sentence about grammatical reasoning that was on lines 657-659.

Overall a very cool study and well done. I think by making the results section clearer this will turn out to be a great paper. It would be cool to see future research at Luminosity minimizing confounders by altering the number of prompts/notifications or changes in the app followed by a regression-discontinuity design.

Interesting idea. The size and nature of the Lumosity program could make it a good testing ground for measuring the impact of the frequency and type of test reminders or notifications. This could be done using regression-discontinuity, as mentioned, or a randomized, parallel "split test" between two product variants. A regression-discontinuity design might also be applicable elsewhere.

In any case, thanks for helping us to improve the paper, especially its clarity with regard to the modeling.

In the interest of transparency, I sign all my reviews the comments made are meant to be constructive and I think that this paper has great promise.

Nicholas Judd, Ph.D.

**Journal**

When submitting your revision, we need you to address these additional requirements.

1. Please ensure that your manuscript meets PLOS ONE's style requirements, including those for file naming. The PLOS ONE style templates can be found at 
https://journals.plos.org/plosone/s/file?id=wjVg/PLOSOne_formatting_sample_main_body.pdf and 
<https://journals.plos.org/plosone/s/file?id=ba62/PLOSOne_formatting_sample_title_authors_affiliations.pdf>

Done.

2.Please provide additional details regarding participant consent. In the ethics statement in the Methods and online submission information, please ensure that you have specified what type you obtained (for instance, written or verbal, and if verbal, how it was documented and witnessed). If your study included minors, state whether you obtained consent from parents or guardians. If the need for consent was waived by the ethics committee, please include this information.

Done. Please see the revised Ethics Statement below.

3. Thank you for stating the following in your Competing Interests section:

"I have read the journal's policy and the authors of this manuscript have the following competing interests: The present study examined effects of cognitive training with Lumosity on the NeuroCognitive Performance Test, both of which are produced by Lumos Labs, Inc.  All authors are current paid employees of the company, and all hold stock in the company."

Please complete your Competing Interests on the online submission form to state any Competing Interests. If you have no competing interests, please state "The authors have declared that no competing interests exist.", as detailed online in our guide for authors at <http://journals.plos.org/plosone/s/submit-now>.

Will do.

This information should be included in your cover letter; we will change the online submission form on your behalf.

Please see the revised Competing Interests Statement below.

4. Thank you for providing the following Funding Statement:

"No external funding contributed to this research; Lumos Labs, Inc. supported the research through the development of its software tools and employment of the authors. The specific roles of these authors are listed in the Author Contributions section. Lumos Labs had no other role in the study design, data collection and analysis, decision to publish, or preparation of the manuscript. Legal approval for publication before submission of the manuscript was obtained from Lumos Labs."

We note that one or more of the authors is affiliated with the funding organization, indicating the funder may have had some role in the design, data collection, analysis or preparation of your manuscript for publication; in other words, the funder played an indirect role through the participation of the co-authors.  If the funding organization did not play a role in the study design, data collection and analysis, decision to publish, or preparation of the manuscript and only provided financial support in the form of authors' salaries and/or research materials, please review your statements relating to the author contributions, and ensure you have specifically and accurately indicated the role(s) that these authors had in your study in the Author Contributions section of the online submission form. Please make any necessary amendments directly within this section of the online submission form.

Lumos Labs provided support in the form of salaries for authors AMO, PIJ, NFN, KRK, and RJS, but did not have any additional role in the study design, data collection and analysis, decision to publish, or preparation of the manuscript.

No changes are required to our prior statements on the authors’ contributions.

Please also update your Funding Statement to include the following statement: “The funder provided support in the form of salaries for authors [insert relevant initials], but did not have any additional role in the study design, data collection and analysis, decision to publish, or preparation of the manuscript. The specific roles of these authors are articulated in the ‘author contributions’ section.”

Done. Please see the revised Funding Statement below.

If the funding organization did have an additional role, please state and explain that role within your Funding Statement.

NA.

Please also provide an updated Competing Interests Statement declaring this commercial affiliation along with any other relevant declarations relating to employment, consultancy, patents, products in development, or marketed products, etc.

Within your Competing Interests Statement, please confirm that this commercial affiliation does not alter your adherence to all PLOS ONE policies on sharing data and materials by including the following statement: "This does not alter our adherence to  PLOS ONE policies on sharing data and materials.” (as detailed online in our guide for authors http://journals.plos.org/plosone/s/competing-interests). If this adherence statement is not accurate and  there are restrictions on sharing of data and/or materials, please state these. Please note that we cannot proceed with consideration of your article until this information has been declared.

Done. Please see the revised Competing Interests Statement below.

5. Please review your reference list to ensure that it is complete and correct. If you have cited papers that have been retracted, please include the rationale for doing so in the manuscript text, or remove these references and replace them with relevant current references. Any changes to the reference list should be mentioned in the rebuttal letter that accompanies your revised manuscript. If you need to cite a retracted article, indicate the article’s retracted status in the References list and also include a citation and full reference for the retraction notice.

Done.

***Revised Ethics Statement***

Both the study and release of the de-identified data were determined by WCG IRB ([www.wcgirb.com](http://www.wcgirb.com/)) to be exempt research under 45 CFR § 46.104(d)(4) because 1) participants’ identities could not be readily ascertained, 2) the investigator did not contact participants, and 3) participants would not be re-identified. Use of de-identified data for any purpose is also covered in the privacy policy agreed to by all participants during their registration to use the Lumosity CT program ([www.lumosity.com/legal/privacy_policy](http://www.lumosity.com/legal/privacy_policy)).  Although the study was determined to be exempt research and participants had already agreed to the use of their deidentified data, participants were additionally informed online immediately prior to the administration of each assessment battery that their data would be used for research purposes.  To proceed with the assessment, participants clicked an “I Agree” button.

***Revised Competing Interests Statement***

I have read the journal's policy and the authors of this manuscript have the following competing interests: The present study examined effects of cognitive training with Lumosity on the NeuroCognitive Performance Test, both of which are produced by Lumos Labs, Inc.  All authors are current paid employees of the company and all hold stock in the company. This does not alter our adherence to PLOS ONE policies on sharing data and materials.

***Revised Funding Statement***

No external funding contributed to this research; Lumos Labs, Inc. funded the research through the development of its software tools and through the employment of AMO, PIJ, NFN, KRK, and RJS. The specific roles of these authors are articulated in the “Author Contributions” section. Other members of the company contributed suggestions and ideas during the design of the study and preparation of the manuscript. Lumos Labs had no other role in the study design, data collection and analysis, decision to publish, or preparation of the manuscript. Legal approval for publication before submission of the manuscript was obtained from Lumos Labs.
