# Supplementary material for: Transfer of learning: Analysis of dose-response functions from a large-scale, online, cognitive training dataset
Source: PLoS One. 2023 May 17;18(5):e0281095. doi: 10.1371/journal.pone.0281095 (PMC10191334; doi:10.1371/journal.pone.0281095)
Supplement: Attachment — Submitted filename: Responses to Editor Reviewer and Journal 12-27-2022.docx [file pone.0281095.s008.docx]

**Editor**

*Authors have covered most of the review comments but still there are some minor edits are required. Go through the review comments and submit the revised version.*

Done. See below.

**Reviewer 2**

*Major points:*

*1) These age brackets seem somewhat arbitrary and the rationale is still not explained, this is key since the main finding is a null of age for Rate and Asymptote. I would like to see all models with age as a continuous variable and education coded ordinally (e.g., 1,2,3,4). These can be put in the SI or just in a response letter.*

We believe that there are two separate points here, each of which we address separately.

**Age brackets.** The first point concerns whether the main findings of the study concerning age depend upon our particular choice of age brackets. These findings are that 1) the effects of repeated testing on the NCPT (measured by the intercept of the exponential fits to D-R functions) diminished with age, while 2) the effects of transfer from CT (measured by the asymptote-intercept and rated parameters) remained constant.

To address this point, we have added a supplementary section (S2 Additional Analyses, referenced on lines 507-8 in the revised paper with tracked changes) containing analyses in which age is treated as a continuous variable. The three ANCOVAs contained in this section correspond to the three main analyses in the paper examining how D-R functions vary with age and reach the same conclusions. We agree that they are a useful addition to the paper.

There are two main reasons we use age brackets in the paper. First, the effects of adult aging on cognitive performance are known to be nonlinear -- and sometimes even nonmonotonic -- within the broad age range studied here. Of particular relevance, Morrison et al. [24] report the relationship between age and the NCPT (which is used in the current analysis) and show that performance improves with age until the mid-twenties, at which point it begins to decline. This relationship is supported in a much larger data set just published by Jaffe et al. [25].

Second, although age is continuous, the impact of age on cognition may be qualitatively different for individuals in different phases of the adult lifespan. As such, studies typically enroll subjects within narrower age ranges (e.g., only older adults), and the use of age bracketing in the literature is common when age ranges are wider. Despite the common use of age brackets, we acknowledge that there is little consensus on the best bracket limits. Given the distribution of ages in our data set, we were able to define sizable bins corresponding roughly to younger adults, middle age, older adults, and the elderly.

Please note that the primary method employed in this study compares the parameters of exponentials fit to participants in different age brackets (see fit models section in Methods). This particular approach requires treating age and other factors as categorical variables. As discussed above, there are legitimate reasons for treating age in this manner. Moreover, as shown in the new supplementary section, the conclusions reached with this method are supported by an alternative form of analysis that does not depend on the use of brackets or on the specific limits of those brackets. And, because the data set is publicly available, we hope that anyone interested in other alternative approaches will be empowered to pursue them

**Educational level as an ordinal variable.** The second point concerns a finding upon which we place much less emphasis (and even acknowledge conflicting findings in the Discussion). This concerns the finding that neither the effects of repeated testing nor transfer varied with educational level. This conclusion is based on two analyses that include educational level as a categorical variable. The first analysis (Table 2) was a preliminary analysis which showed no effect of educational level on change in NCPT GI. This suggests that the D-R function does not vary across educational level. More definitive evidence for this conclusion was presented by the second analysis (Table 3), which involved our primary approach of comparing parameters of fit exponentials. Here we found the same intercept, rate, and asymptote-intercept at all four levels of education. However, as mentioned above, this approach requires that all factors be categorical. So the effects of education as an ordinal variable are examined here in a set of analyses based on the linear regression presented in Table 2. In line with your Comment #1, we have chosen to just include them in this response letter.

The first of these analyses is shown in the table below. (For a version with properly aligned rows, please see the attached file containing our responses.) Here, educational level is treated as an ordinal variable (with levels coded as 1,2,3, and 4), age is treated as a continuous variable rather than a categorical one (we thought you would prefer this), and gender is still categorical. As you can see there are three terms associated with educational level. This is because R deals with ordinal variables by fitting polynomials to the integer values of the levels (here four) and providing a separate term for each coefficient (here linear, quadratic, and cubic). None of the educational terms were significant. In contrast, as in Table 2, those for age and log of the number of gameplays between NCPT assessments were highly significant.

| **Model: GI_change ~ log1p(num_games) + con_age + ord_edu + gender** | | | | | |
| --- | --- | --- | --- | --- | --- |
| **Coefficients:** | **Estimate** | **Std. Error** | | **t Value** | **Pr(> \|t\|)** |
| Intercept  (0 gameplays, 0 yo, HS, F) | 0.9225 | | 0.1291 | 7.147 | 8.96e-13*** |
| Log1p(gameplays) | 0.7040 | | 0.0190 | 37.136 | < 2e-16*** |
| Con_Age | -0.0235 | | 0.0015 | -15.752 | < 2e-16*** |
| Ord_Edu_L | 0.0792 | | 0.0563 | 1.407 | 0.159 |
| Ord_Edu_Q | 0.0018 | | 0.0503 | 0.035 | 0.972 |
| Ord_Edu_C | -0.0599 | | 0.0435 | -1.378 | 0.168 |
| Gen (M) | 0.0278 | | 0.0467 | 0.594 | 0.552 |

Notes. Con_Age is continuous, Ord_Edu is ordinal, and Gen is categorical. L, Q, and C refer respectively to the linear, quadratic, and cubic terms of a polynomial fit to the four-levels of Ord_Edu.

The other analyses are all variations of the above one. First, to produce a single term for educational level, we treated this variable as continuous (still with levels 1-4), while keeping everything else the same. This did produce a trend towards significance (t = 1.704, p = 0.088). But, the effects for both the continuous (t = 1.267, p = 0.205) and ordinal (t’s = 1.014, -0.002, -1.392; p’s = 0.310, 0.999, 0.164) versions of educational level were far from significant when we replaced the continuous version of age with a categorical one (which was even more significant when categorical). Perhaps this is due to the categorical version of age (which captures nonlinear changes in cognitive performance) accounting for more variance shared with educational level than the continuous (linear) version. But, in any case, these analyses provide little evidence that the D-R function varies with educational level (or gender), either via the effects of repeated testing or transfer. Rather, they support our emphasis in the paper on age, which did produce conclusive and interpretable effects.

*2) The biggest limitation in this study (which you do highlight in the discussion) is the lack of independence of dose. While I think it is acceptable, you need to show how much of an issue it is. I think this should be empirically tested; Dose ~ GI_unnormedTimePoint1 + age + edu + gender. This could also be done with GI change.*

We agree that the lack of randomization in an observational study like this one is a limitation (as described in the Discussion), but we disagree that differences in dose across demographic groups is a limitation. This latter issue received considerable attention in the paper.

First, we did test for differences in dose between demographic groups, reported the existence of such differences, and examined how D-R functions vary across demographic groups when differences in dose are controlled for. This information is provided in the paragraph immediately preceding Table 2.

“First, to see whether D-R functions differed in any respect across each demographic factor, we performed a multiple linear regression that compared the overall amount of change in GI between demographic levels, while controlling for differences in amount of gameplay. Amount of gameplay did, in fact, differ across the levels of each demographic factor. An ANOVA (with Type 2 SSs to control for correlations between factors) found significant effects on number of games played of Age (F(3,93554) = 74.012, p < 0.0001), Education (F(3,93554) = 32.611, p < 0.0001), and Gender (F(1,93554) = 215.512, p < 0.0001). The linear regression of demographic effects on overall change in GI therefore included the number (on a log scale) of each participant’s gameplays between T1 and T2 as a covariate. The results are shown in Table 2. As can be seen, overall change in GI between T1 and T2 differed between Age levels (F(3,93553) = 79.592, p < 0.0001), but not between levels of Education (F(3,93553) = 1.221, p = 0.3003) or Gender (F(1,93553) = 0.626, p = 0.4287). Note also the significant (p < 0.001) increase in GI change scores with number of games.”

We acknowledge that the wording of the above paragraph makes it difficult to discern two separate analyses: 1) the analysis showing the existence of dose differences between demographic groups (middle of paragraph), and 2) the preliminary analysis of demographic effects on D-R functions while controlling for these dose differences (beginning and end of paragraph). We have therefore tried to improve the wording (lines 421-3 in the revised paper with tracked changes).

Second, in the final paragraph of the Results subsection containing the analyses reported in Tables 2 and 3 (“Comparison of D-R functions across age, education, and gender”), we conclude the following:

"The above two analyses provide converging evidence that the positive relation between dose and response cannot be explained by demographic differences between individuals with different amounts of training, as this positive relation occurs at each combination of demographic levels. Both analyses found change in GI (response) to be positively related to amount of CT (dose). Neither found significant differences between the D-R functions across different levels of education or gender. The differences in GI change found across age in the linear regression were shown in the exponential analysis to result solely from differences in the intercept of D-R functions, rather than from differences in asymptote – intercept or rate, thus preserving the positive relation between dose and response.”

Finally, as you mentioned, possible problems arising from a lack of independence between dose and other factors are acknowledged in the Discussion (Limitations). “The present study has a number of limitations. Some stem from its observational and cross-sectional design.  Each dose level in the same D-R function involved a different set of participants, and these participants were not randomly assigned to that level.  Rather, each participant decided upon their own dose, i.e., the amount of CT they engaged in between the two NCPT assessments.  This leaves open the possibility that participants with different doses differed in other ways that influenced the amount of change on the NCPT.” However, we then state “Despite that, the positive relation between dose and response cannot be explained by demographic differences between individuals with more or less training, as this relation occurred at each level of age, education, and gender.”

*Minor points:*

*- Figure 7 derived from “Age differences in D-R functions” is very nice and should stay in the main text. For clarity to the reader, I would recommend putting everything else – including the table in the SI as it is just a simpler model (i.e., Model 2) than the preceding one and the results do not change. I see this section as just a nice way to make Figure 7. If you insist on keeping it in the main text the order of results should match the methods (alternatively you could then put Model 3 in the supp. And add a since sentence “when adjusting for education and gender the results did not change (see SI Table X)”.*

We are happy to hear the positive feedback on Figure 7 and appreciate the suggestion for moving (or removing) portions of the results that are seen as supportive but not critical. We believe this is a stylistic preference, and respectfully disagree that it would be an improvement to the paper. We believe that all the material presented in this section is important in order to present, quantify, evaluate statistically, and interpret the pattern of results illustrated by Figure 7. So we would prefer to keep the section as is.

*- Also please remove the sentence about “To increase power…” (line 462) since this is not necessarily true – those extra covariates could be reducing residual error thereby increasing power.*

OK. We removed “To increase power,” but left the rest of the sentence. It now reads, “We therefore combined the different levels of education and gender and examined how the parameters of fit exponentials varied across the sole factor of age.”

*- Line 279 should be slightly rewritten to clarify it is multiple – i.e., 3 – dummy variables, as some readers might be less familiar with dummy coding. Later, explanations were handled in a clearer fashion.*

Done. (See line 279 in the revised paper with tracked changes).

*- This whole section was a very good addition.*

Thanks! We wrote it to address the major comment in your previous review and think that it improved the paper considerably.

*- I really like Figure 3*

Thanks. We think this figure gives a nice overall picture of the effects of training – both before and between NCPT assessments - and that our response to Rev 1 helped make it accessible to most readers.

*- Line 753-754 is not necessarily true, as each group could have different levels of motivation (or any other unobserved confounder). For example, there could be real differences between age groups yet motivation is equally counteracting in a continuous fashion across groups – leading to a null finding.*

We think that you are referring to the sentence “Conversely, the relation between chosen dose and motivation or expectations would need to be the same across each demographic factor.” This sentence was meant to express an implication of the hypothesis that the positive relation between dose and change on NCPT could be due solely to participants with greater doses being more motivated or having greater expectations of improvement. But, after considering the above comment, we no longer think that this is necessarily the case. So we have changed this and the preceding sentence to the following (see italics).

“… While it cannot be conclusively rejected, an explanation attributing all positive D-R relations found in the present study to motivation or expectations would be far from parsimonious. *Such an explanation would need to account for both the differences between subtests and lack of differences between demographic groups in the amount that change on the NCPT increased with dose.* Concerns about motivation or expectations in future studies with designs like ours might be addressed through self-reports or questionnaires.”

In closing, we would like to mention that, if our paper is published, we plan to include its peer review history. Since the dataset is public, readers should be able to further investigate issues related to reviewers’ comments or to pursue questions of their own. Indeed, we would welcome additional analyses of this dataset by other researchers.

Thank you very much for all your time and effort.

**Journal Requirements**

12-24-2022

*Please review your reference list to ensure that it is complete and correct. If you have cited papers that have been retracted, please include the rationale for doing so in the manuscript text, or remove these references and replace them with relevant current references. Any changes to the reference list should be mentioned in the rebuttal letter that accompanies your revised manuscript. If you need to cite a retracted article, indicate the article’s retracted status in the References list and also include a citation and full reference for the retraction notice.*

The references have been checked and are now formatted in Vancouver style. We also added an additional reference [25] and replaced two others with new ones ([27] and [29] in revision). This was done to improve the background information provided about the neuropsychological tests employed in the study.

Please be aware that, in addition to the revised manuscript, we are submitting a revised version of S2_appendix and a new file entitle “S2_additional_analyses.”

12-27-2022

The most recent revision of our paper was submitted to PLOS One on 12-24-2022. On 12-26-2022, the paper was sent back to the authors with the following queries and requests for further information from the Journal about the Funding/Financial Disclosure Statement.

*We've checked your submission and before we can proceed, we need you to address the following issues:*

*1.* *Thank you for stating the following financial disclosure: [No external funding contributed to this research;* *Lumos Labs, Inc. funded the research through the development of its software tools and through the employment of AMO, PIJ, NFN, KRK, and RJS. The specific roles of these authors are articulated in the “Author Contributions” section.* *Other members of the company contributed suggestions and ideas during the design of the study and preparation of the manuscript. Lumos Labs had no other role in the study design, data collection and analysis, decision to publish, or preparation of the manuscript. Legal approval for publication before submission of the manuscript was obtained from Lumos Labs]*

*At this time, please address the following queries:*

*a) Please clarify the sources of funding (financial or material support) for your study. List the grants or organizations that supported your study, including funding received from your institution.*

This is already done in the previously provided statement above:

“No external funding contributed to this research; Lumos Labs, Inc. funded the research through the development of its software tools and through the employment of AMO, PIJ, NFN, KRK, and RJS.”

*b) State what role the funders took in the study. If the funders had no role in your study, please state: “The funders had no role in study design, data collection and analysis, decision to publish, or preparation of the manuscript.”*

This is already done in the previously provided statement above:

“Other members of the company contributed suggestions and ideas during the design of the study and preparation of the manuscript. Lumos Labs had no other role in the study design, data collection and analysis, decision to publish, or preparation of the manuscript.”

*c) If any authors received a salary from any of your funders, please state which authors and which funders.*

This is already done in the previously provided statement above:

“Lumos Labs, Inc. funded the research through … the employment of AMO, PIJ, NFN, KRK, and RJS.”

Please see also the previously provided Competing Interests statement, which includes:

“All authors are current paid employees of the company and all hold stock in the company.”

*d) If you did not receive any funding for this study, please state: “The authors received no specific funding for this work.”*

N/A

*Please include your amended statements within your cover letter; we will change the online submission form on your behalf.*

We are unable to see how any amendments are necessary or what additional information satisfy queries a-d above.

Please note that the previously provided Financial Disclosure Statement is almost identical (except for the authors and some minor details) to one that appeared in a previously published paper by our company (Lumos Labs, Inc.) in PLOS One:

<https://journals.plos.org/plosone/article?id=10.1371/journal.pone.0134467>

*2. We note that the information you provided in the ‘Funding Information’ and ‘Financial Disclosure’ sections do not match.*

We are not sure what is meant by “Funding Information and Financial Disclosure sections.” There are not separate Funding Information and Financial Disclosure statements. So, by “sections”, presumably you mean parts of the same statement. The funding and financial disclosure information in the previously provided Funding/Financial disclosure statement above are in agreement. This statement is also in agreement with the Competing Interests statement.

*When you resubmit, please ensure that you provide the updated Funding Information.*

For the reasons discussed above, there is nothing to provide. We have no idea what additional information is being requested or what could be missing. Please compare our Funding Statement with that in a paper previously published by our company in the Journal (link provided above).
